# Hypertensive Emergency as the Initial Presentation of Acquired Thrombotic Thrombocytopenic Purpura: A Case Report

**DOI:** 10.7759/cureus.20176

**Published:** 2021-12-05

**Authors:** Diana I Zamora, Laura M Gonzalez

**Affiliations:** 1 General Medicine, Universidad de Ciencias Medicas, San Jose, CRI

**Keywords:** adamts13 protein, purpura, malignant hypertension, thrombotic thrombocytopenic purpura, thrombotic microangiopathies

## Abstract

Thrombotic thrombocytopenic purpura (TTP) is a type of thrombotic microangiopathy (TMA) related to a deficiency of ADAMTS13 protein, which could lead to fatal outcomes. TTP presents a vast array of symptoms, making its diagnosis a challenge to physicians. In this report, we discuss the case of an 80-year-old male who presented with abdominal pain and hypertension with underlying severe thrombocytopenia and hemolysis. Although his presentation could also be secondary to malignant hypertension, he was promptly diagnosed with TTP based on these findings. TTP is a hematologic emergency, and it should be kept in the differential diagnosis when a patient presents with thrombocytopenia and hemolysis with or without accompanying symptoms.

## Introduction

Thrombotic thrombocytopenic purpura (TTP) is a rare and highly fatal disease. It forms part of thrombotic microangiopathies (TMAs) and is characterized by hemolytic anemia, thrombocytopenia, and multiorgan dysfunction due to microthrombosis in arterioles and capillaries [1]. TTP can present with a vast array of symptoms such as petechiae, neurological deficits, confusion, seizures, and even myocardial infarction. It is not uncommon to find abdominal pain as the primary concern of patients looking for medical attention, such as in the case presented here. However, not all patients with TTP are critically ill. The majority of patients do not present with clear symptoms of thrombocytopenia when they seek medical attention; they are usually diagnosed incidentally following a routine complete blood count [2]. The PLASMIC score [*P*latelet count; combined hemo*L*ysis variable; absence of *A*ctive cancer; absence of *S*tem-cell or solid-organ transplant; *M*ean corpuscular volume (MCV); *I*nternational normalized ratio (INR); *C*reatinine] is key to establishing a prompt and accurate diagnosis in a patient with thrombocytopenia and hemolytic anemia. The TTP pentad is only present in 10% of patients and it is unreliable to use this as the sole parameter for the diagnosis. This pentad consists of fever, neurological abnormalities, kidney injury, abdominal pain, and thrombocytopenia [3]. Due to the ample and varied presentation of TTP, these parameters will aid in differentiating it from other causes of microangiopathies such as hypertensive emergencies that can present initially in a similar manner. In this report, we aim to highlight the crucial need for accurate differentiation between TTP and hypertensive emergency, as well as emphasize the importance of rapid management to reduce fatality in this type of hematologic emergency.

## Case presentation

An 80-year-old Indian male presented to the emergency department (ED) with a one-day history of epigastric and left lower quadrant pain with mild relief after ibuprofen administration. The patient had a history of hypertension, diabetes mellitus type 2, hyperlipidemia, prostate cancer, and subclinical hypothyroidism. His surgical history included an endoscopic retrograde cholangiopancreatography (ERCP) and laparoscopic cholecystectomy. His medications at that time included metformin, omeprazole, gabapentin, carvedilol, atorvastatin, ferrous sulfate, cyanocobalamin, levothyroxine, amlodipine, guaifenesin, and aspirin. The patient disclosed that he was inconsistent with taking his medications and stated that his conditions were poorly controlled. He denied any alcohol, tobacco, or recreational drug use. Vital signs on admission evidenced high blood pressure of 182/95 mmHg, a pulse of 89 bpm, a temperature of 36.4 °C, respiratory rate of 19, and mildly low oxygen saturation of 92% on room air. There were no abnormal findings on cardiopulmonary examination. Associated symptoms included shortness of breath, nausea, and hematuria. On examination, the abdomen was soft, non-distended, but was tender to palpation in the epigastric and left lower quadrant region. Rectal examination showed no masses, a small amount of stool in the vault, and a guaiac exam was negative. There were no ecchymoses or signs of active bleeding at the time of examination. The patient was awake and oriented to person, place, and time, and he spoke coherently. He had no focal neurological deficits. On further evaluation, he indicated that he had visited a primary care clinic with similar symptoms the day before. He had presented to the primary care clinic with a blood pressure of 168/80 mmHg. He had then been administered 324 mg of aspirin, nitroglycerin, and 4 mg of ondansetron that resulted in minimum symptom relief. Electrocardiogram (EKG) at the ED showed normal sinus rhythm and the rate was within normal limits. Cardiac ultrasound showed a grossly normal heart with good global function. Troponin I level was 0.124 ng/ml (reference value: <0.04 ng/ml); urinalysis showed evidence of proteinuria (+++), macroscopic hematuria, and red blood cells of 4-20/hpf. Initially, the patient was treated as a case of hypertensive emergency due to the presence of hematuria, proteinuria, epigastric pain, and elevated blood pressure. He was administered 10 mg IV hydralazine, 5 mg amlodipine, and 40 mg atorvastatin.

Approximately six hours after the patient presented to the ED, he became obtunded, and his temperature dropped to 34.6 °C, which prompted an evaluation for sepsis. A complete blood count revealed severe anemia [hemoglobin: 8.7 g/dL (normal range: 13.5-17.5 g/dL)] and thrombocytopenia [platelets: 11,000/mm^3^ (150,000-400,000/mm^3^)], reticulocyte count of 1.9% (0.5-1.5%), with a prothrombin time of 13 seconds and partial thromboplastin time of 27 seconds. A blood smear showed normocytic normochromic anemia with increased red cell regeneration and occasional red cell fragments. Supportive laboratory results demonstrated hyperbilirubinemia of 3.6 mg/dL (0.1-1.0 mg/dL), with a predominance of unconjugated bilirubin of 3.3 mg/dL (0.3-1.0 mg/dL), direct bilirubin of 0.3 mg/dL (<0.3 mg/dL), elevated lactate dehydrogenase (LDH) of 295 U/L (45-90 U/L), haptoglobin undetectable (50-220 mg/dL). Blood urea nitrogen (BUN) of 16 mg/dL (7-30 mg/dL) and creatinine of 0.8 mg/dL (0.7-1.2 mg/dL) were observed. Blood cultures were negative at that time. After reviewing the laboratory findings, Hematology was consulted for possible splenic sequestration, and the patient was given one unit of platelets for the prevention of intracranial hemorrhage. One hour post-transfusion, the patient became hypertensive again (193/86 mmHg), febrile (38 °C), aphasic, and agitated. On neurological examination, he had a Glasgow Coma Scale (GCS) score of 8 and had a sluggish right pupil; petechiae on bilateral upper and lower extremities were present at that time but there were no active bleeding sites. He was taken for a CT of the head for which he required sedation, and secondary to this, he desaturated to SpO_2_ of 70% on room air and was subsequently intubated. CT head revealed no acute intracranial pathology. Repeat laboratory values showed a decrease in platelets to 6,000/mm^3^. At this time, Hematology advised to admit the patient to the ICU and perform an ADAMTS13 activity assay level since the patient had presented the five findings that make up the rare pentad of TTP: fever, neurological symptoms, thrombocytopenia, abdominal pain, and renal insufficiency. Hematology also advised starting plasmapheresis exchange therapy (PEX) immediately because of the critical nature of the illness, as well as administering 1 mg/kg daily of prednisone after daily PEX.

The patient was treated as advised with marked improvement. ADAMTS13 level came back critically low five days later: <5% activity (normal range: 50-160% activity, confirmatory level: <10%), which confirmed the diagnosis. PEX therapy and prednisone were continued for a total of eight days until platelet levels reached >150,000/mm^3^ for two days and LDH levels returned to normal. Glucocorticoids were tapered off when platelet count remained above 150,000/mm^3^. At the time of discharge, relevant laboratory values revealed platelets of 171,000/mm^3^, LDH of 65 U/L, haptoglobin of 84 mg/dL, and hemoglobin of 10.1 g/dL. The patient was discharged approximately 10 days after the ICU admission with low-dose rituximab once per week for four weeks as a preventative treatment for relapse and a follow-up appointment in the Hematology clinic to monitor ADAMTS13 activity assay.

## Discussion

We discussed the case of a patient with acquired TTP who presented with and was initially treated as a case of hypertensive emergency. TTP is a rare and fatal hematological disease, with an annual prevalence of approximately 10 cases/million people and an annual incidence of one new case/million people [4]. Acquired TTP, as seen in this patient, is caused by the presence of autoantibodies against ADAMTS13. This metalloprotease is essential in the cleaving process of the von Willebrand factor (VWF). Without ADAMTS13, ultra-large VWF multimers with bounded platelets persist in the bloodstream, leading to thrombocytopenia. This leads to the accumulation of microthrombi in the capillaries that in turn lead to hemolysis. This also affects the blood flow in the microcirculation that causes end-organ damage, mainly in the central nervous, renal, and cardiovascular systems [5]. TMA in a hypertensive emergency is secondary to shear stress due to the narrowing of vessels that leads to excessive fragmentation of erythrocytes and the consumption of platelets [6]. Because of the difference in pathogenesis, the thrombocytopenia seen in TTP is much more severe (platelets of <15,000/mm^3^) than the one seen in malignant hypertension (platelets of around 60,000/mm^3^​​). Also, renal function in TTP is not as affected as in malignant hypertension. Renal manifestations in TTP are hematuria and proteinuria, whereas renal manifestations in malignant hypertension include a rise in creatinine, BUN, hematuria, and proteinuria [4].

There are two types of acquired TTP: idiopathic and non-idiopathic. Non-idiopathic TTP is seen in about 50% of patients diagnosed with this disorder. The most frequent conditions associated with TTP, serving as triggers, are as follows: autoimmune disorders (e.g., systemic lupus erythematosus), viral and bacterial infections, cancer, pregnancy, and drugs (e.g., cyclosporine, quinine, clopidogrel). Idiopathic TTP is seen in the remaining 50% of cases [4].

Diagnosing TTP presents a challenge to physicians since it has an ample array of symptoms and they may not be all evident in the initial consultation. The gold standard in diagnosing TTP is the ADAMTS13 activity assay. If its activity is less than 10%, it is diagnostic for TTP. Severe thrombocytopenia and hemolysis must also be present to confirm the diagnosis of TTP. A helpful tool in the prompt diagnosis of TTP when ADAMTS13 activity assay is not readily available is the PLASMIC score. It has been shown to be useful in the accurate diagnosis and in predicting the prognosis of TTP (Table 1). A PLASMIC score equal to or greater than 6 is highly predictive of ADAMTS13 deficiency and necessitates immediate treatment [4]. The key differences between TMA secondary to hypertension and acquired TTP are as follows: TMA secondary to malignant hypertension will be seen with extreme hypertension (diastolic blood pressure of >130 mmHg), a past medical history of uncontrolled hypertension, clear renal dysfunction, and moderate thrombocytopenia. A patient with TTP that presents with hypertension will not have retinal findings and will also not recover from thrombocytopenia after the management of hypertensive crisis [7].

**Table 1:** Modified PLASMIC score LDH: lactate dehydrogenase; MCV: mean corpuscular volume; INR: international normalized ratio

The PLASMIC score - modified	Points
Platelet count <30,000/10^9^/L	1
Hemolysis variables present (increased reticulocytes, increased LDH, decreased haptoglobin, increased indirect bilirubin)	1
No active cancer	1
No history of solid organ or stem cell transplant	1
MCV <90 fl	1
INR <1.5	1
Creatinine <2.0/mg	1

A presumptive diagnosis is made based on the presence of microangiopathic hemolytic anemia and thrombocytopenia. Even though the presence of severe deficiency of ADAMTS13 activity or an inhibitor to it confirms the diagnosis of acquired TTP, it is not essential for starting prompt treatment [8]. TTP is a medical emergency, and PEX remains the treatment of choice and should be started as soon as possible. Plasma exchange has been associated with a decrease in mortality from 80-90% to 10%. During this procedure, plasma is removed from the affected patient and replaced by a donor’s plasma. In the course of the therapy, autoantibodies and immune complexes are removed from the affected patient’s plasma, and ADAMTS13 and VWF-multimers analysis (MM) of normal composition are replaced [5]. PEX is performed daily until features of organ involvement are resolved, platelet count has recovered to levels >150,000/mm^3^ for two consecutive days, and hemolysis has stopped (seen in decreased levels of LDH). Immunosuppression with corticosteroids (methylprednisolone 1 mg/kg/day) is frequently used along with PEX, in order to control the autoimmune response and to improve the efficacy of plasma exchange therapy. Prophylactic platelet transfusion can worsen the symptoms of TTP and therefore should be reserved for severe cases [9].

Rituximab, a monoclonal antibody, is usually added to the therapy regimen in patients with refractory TTP, given its benefit in suppressing the production of IgG antibodies against ADAMTS13, with improved platelet recovery and lower relapse rate (relapse defined as thrombocytopenia after more than 30 days of stopping PEX). The addition of rituximab has been shown to induce remission in patients for more than 19 months [10]. New therapeutic approaches are being developed, such as an anti-VWF agent called caplacizumab, to be used subcutaneously, which causes suppression of VWF for up to 48 hours. Several studies have demonstrated reduced time to platelet normalization, exacerbations, and relapse rates [9].

## Conclusions

TTP is a life-threatening hematologic disorder that requires prompt diagnosis and immediate treatment with PEX and maintenance treatment with rituximab to decrease the chances of relapses. Although patients with TTP can present with hypertensive-like crises, it is imperative not to delay treatment with PEX. Using diagnostic parameters such as the PLASMIC score can aid in differentiating between these two conditions. Due to the uncommon nature of the illness and a lack of physician awareness, patients are often not promptly diagnosed, which can negatively affect their prognosis.
